# Effect of genetic polymorphisms rs2301113 and rs2057482 in the expression of HIF-1α protein in periodontal ligament fibroblasts subjected to compressive force

**DOI:** 10.1590/1678-7757-2022-0151

**Published:** 2023-05-29

**Authors:** Erika Calvano KÜCHLER, Vinicius Broska TEODORO, Agnes SCHRÖDER, Ute NAZET, Michelle Nascimento MEGER, Patricia Valéria Manozzo KUNZ, Flares BARATTO-FILHO, Gerrit SPANIER, Rafaela SCARIOT, Peter PROFF, Christian KIRSCHNECK

**Affiliations:** 1 University of Regensburg Department of Orthodontics Germany University of Regensburg, Department of Orthodontics, Germany.; 2 Curitiba Brasil Private Practice, Curitiba, Brasil.; 3 Universidade Tuiuti do Paraná Curitiba Brasil Universidade Tuiuti do Paraná, Curitiba, Brasil.; 4 University of Regensburg Department of Maxillofacial Surgery Germany University of Regensburg, Department of Maxillofacial Surgery, Germany.; 5 Universidade Federal do Paraná Departamento de Estomatologia Curitiba Brasil Universidade Federal do Paraná, Departamento de Estomatologia, Curitiba, Brasil.

**Keywords:** Hypoxia-inducible factor, Tooth movement, Gene

## Abstract

**Objective:**

Many genes and signaling molecules are involved in orthodontic tooth movement, with mechanically and hypoxically stabilized HIF-1α having been shown to play a decisive role in periodontal ligament signaling during orthodontic tooth movement. Thus, this *in vitro* study aimed to investigate if genetic polymorphisms in *HIF1A* (Hypoxia-inducible factor α-subunits) influence the expression pattern of HIF-1α protein during simulated orthodontic compressive pressure.

**Methodology:**

Samples from human periodontal ligament fibroblasts were used and their DNA was genotyped using real time Polymerase chain reaction for the genetic polymorphisms rs2301113 and rs2057482 in *HIF1A* . For cell culture and protein expression experiments, six human periodontal ligament fibroblast cell lines were selected based on the patients’ genotype. To simulate orthodontic compressive pressure in fibroblasts, a 2 g/cm^2^ force was applied under cell culture conditions for 48 hours. Protein expression was evaluated by Western Blot. Paired t-tests were used to compare HIF-1α expression with and without compressive pressure application and unpaired t-tests were used to compare expression between the genotypes in rs2057482 and rs2301113 (p<0.05).

**Results:**

The expression of HIF-1α protein was significantly enhanced by compressive pressure application regardless of the genotype (p<0.0001). The genotypes in the genetic polymorphisms rs2301113 and rs2057482 were not associated with HIF-1α protein expression (p>0.05).

**Conclusions:**

Our study confirms that compressive pressure application enhances HIF-1α protein expression. We could not prove that the genetic polymorphisms in *HIF1A* affect HIF-1α protein expression by periodontal ligament fibroblasts during simulated orthodontic compressive force.

## Introduction

The periodontal ligament (PDL) is a connective tissue attachment located between the cementum of teeth and the alveolar bone. The main PDL cell population comprises fibroblast-like cells characterized by collagen production, but also presenting some osteoblastic features.^1^ Fibroblasts are the predominant cells in the PDL and are responsible for the regulation of tissue homoeostasis and the formation of collagenous structural proteins. These cells are also crucial during orthodontic tooth movement (OTM), which is characterized by the application of mechanical force to a tooth by removable or fixed orthodontic appliances.^2^ During orthodontic treatment, OTM leads to the formation of tensile and pressure zones in the human periodontal ligament.^3^ PDL fibroblasts react to a continuous mechanical pressure with alterations in the expression of many genes important for the regulation and mediation of OTM.^4 - 9^

The hypoxia-inducible factor α-subunits (HIFα) are key transcription factors in the mammalian response to oxygen deficiency.^10^ HIFα acts as a master regulator of cellular and systemic homeostatic response to hypoxia by activating the transcription of many genes important for OTM, including those involved in angiogenesis and apoptosis. Interestingly, this transcription factor has been highlighted as an important molecule during OTM.^8 , 11^ During OTM, a disruption in PDL vascular circulation occurs at pressure areas of the periodontal ligament due to the compression of blood vessels, leading to hypoxic conditions (reduction in the oxygen supply) in the PDL, stabilizing HIF-1α.^12^ Moreover, a recent *in vitro* study showed that HIF-1α protein levels in PDL fibroblasts were predominantly elevated by simulated orthodontic compressive pressure, suggesting that the main stimulus is mechanical and not hypoxia.^8^

The subunit HIF-1α is encoded by the gene *HIF1A,* which was assigned to the human chromosome 14q21–q24. The *HIF1A* gene presents some genetic polymorphisms that have been studied in different health conditions and were associated with cancer susceptibility and prognosis, coronary artery disease, and metabolic and cardiovascular risk factors.^13 - 18^ Genetic polymorphisms in *RANKL* (receptor activator of nuclear factor-κB ligand) and *COX2* (cyclooxygenase-2) genes associated with OTM were shown to affect the expression of those genes in human PDL (hPDL) fibroblasts during simulated orthodontic compressive pressure. PDL-fibroblast from individuals with specific genotypes in *RANKL* and *COX2* responded differently to *in vitro* simulated orthodontic compressive pressure.^9^ Thus, it is possible to hypothesize that genetic polymorphisms in *HIF1A* are involved in interindividual differences in HIF-1α levels as a response to OTM. In this *in vitro* study, we investigated if the genetic polymorphisms rs2301113 and rs2057482 in *HIF1A* gene influence the expression patterns of HIF-1α protein during simulated orthodontic compressive pressure on hPDL fibroblasts.

## Methodology

### Ethical aspects

All experiments were performed in accordance with applicable guidelines and regulations. The ethics committee of the University of Regensburg, Germany, approved the samples collection and experiment (# 12-170-0150). Written consent was obtained from all patients.

**Figure 1:**
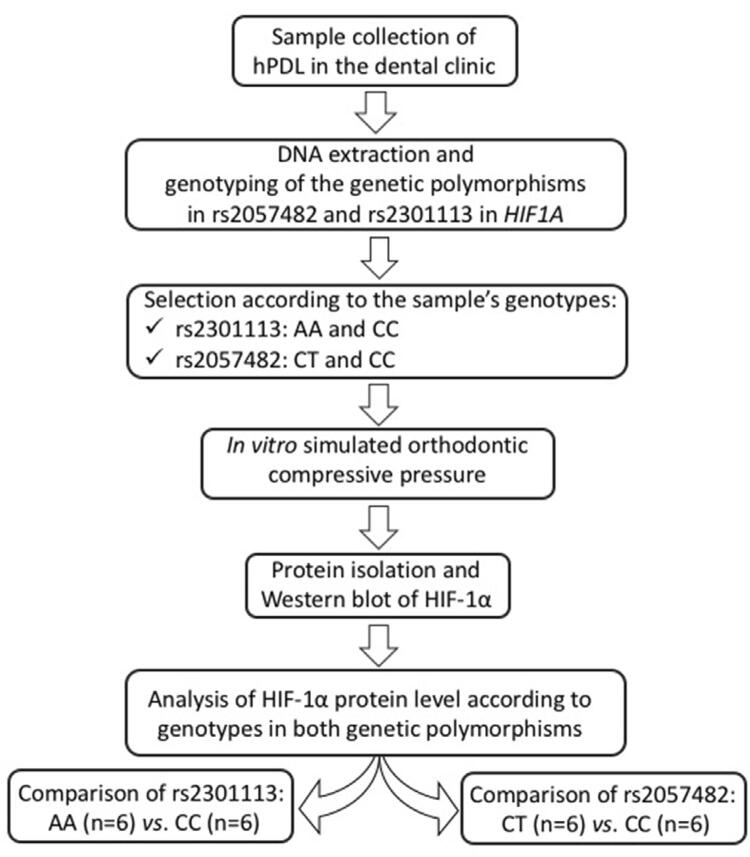
Flow diagram of the experimental design

Figure 1 shows the flow diagram of the experimental design.

### Sample collection and selection, DNA isolation, and allelic discrimination analysis

PDL samples from 57 patients undergoing dental treatment at the maxillofacial surgery clinic at the University of Regensburg were collected.

Caries-free third molars without periodontal disease extracted during dental treatment were used to collect primary human periodontal ligament (hPDL) fibroblasts from periodontal connective tissue. hPDL samples were collected, isolated, cultivated, and characterized according to an established method and protocol.^5 , 6^

The samples of hPDL fibroblasts for cell culture and protein analysis were selected according to the patients’ genotype. Genomic DNA for the allelic discrimination analysis was extracted from these cells. Briefly, the DNA of hPDL cell samples was isolated using the GenElute Mammalian Genomic DNA Miniprep kit (Sigma Aldrich, Munich, Germany). DNA extraction was performed according to the manufacturer’s instructions, as previously described.^19^ The two polymorphisms in *HIF1A* were genotyped with allelic discrimination real-time Polymerase chain reaction (PCR) using the TaqMan assay and the Mastercycler^®^ ep realplex-S thermocycler (Eppendorf AG, Hamburg, Germany), as previously described.^20^Table 1 shows the characteristics of the genetic polymorphisms.

**Table 1:** Characteristics of the studied genetic polymorphisms

Acronym	Name	Genetic Polymorphism	Region	Base Change	Frequency of the polymorphic homozygous in Europeans#
*HIF1A*	*Hypoxia inducible factor 1-alpha*	rs2057482	UTR-3	C/T	0.017-0.040 (TT)
		rs2301113	Intron	A/C	0.16-0.017 (CC)

Note:# https://www.ncbi.nlm.nih.gov/projects/SNP/snp_ref.cgi?do_not_redirect&rs=rs2057482 and https://www.ncbi.nlm.nih.gov/projects/SNP/snp_ref.cgi?do_not_redirect&rs=rs2301113

#### In vitro-simulated orthodontic compressive pressure

For the cell culture experiment, six hPDL cell lines were selected based on their genotype. To simulate orthodontic compressive pressure in hPDL pressure areas, a 2 g/cm^2^ physiological compressive force was applied to the hPDL fibroblasts under cell culture conditions at 70% confluency for 48 h, using a glass disc according to a previously established and published method for simulating orthodontic compressive forces.^5 , 6 , 9^ At the same time, hPDL fibroblasts were cultivated for 48 h without compressive forces (control).

## Protein isolation and Western blot of HIF-1α

The total protein from hPDL was isolated with 100 µL of CelLytic™ M per well (C2978; Sigma-Aldrich^®^) and proteinase inhibitors (TD263654, Thermo Scientific). Protein concentration was determined with RotiQuant (K015.3; Carl Roth GmbH & Co. KG), according to the manufacturer’s instructions. For immunoblotting, 8% SDS-polyacrylamide gel under reducing conditions was used, and proteins were transferred to the polyvinylidene difluoride (PVDF) membranes via electroblotting. The membranes were blocked with 5% nonfat milk in Tris-buffered saline and 0.1% Tween 20, pH 7.5 (TBS-T), for one hour at room temperature. For protein detection, the membranes were incubated with anti-HIF-1α (1:2 000, 10006421, Cayman Chemical, Michigan, USA) and anti-β-actin (reference, 1:5 000, ABIN274248, antibodies-online GmbH, Aachen, Germany) over night at 4°C. After washing three times in TBS-T, the blots were incubated at room temperature for one hour with horseradish peroxidase-conjugated anti-rabbit IgG (611-1302, Rockland Immunochemicals, Inc. Limerick, Pennsylvania, USA) diluted 1:5 000 in 0.5% milk in TBS-T. The antibody binding was observed with an enhanced chemiluminescence system (WBLUF0100, Millipore, Merck KGaA, Darmstadt, Germany) and bands quantified densitometrically with ImageJ (ver. 1.47, Wayne Rasband, National Institutes of Health, USA).

## Statistical analysis

For the genetic polymorphism rs2301113, homozygotic samples with AA (carrying two mutant alleles) and CC (carrying two wild alleles) were selected for the comparison: AA *versus* CC. For the genetic polymorphism rs2057482, homozygotic samples from patients carrying two mutant alleles were not observed, thus heterozygotic samples with CT (carrying one mutant allele) and homozygotic samples with CC (carrying two wild alleles) were selected for the comparison: CT *versus* CC.

The software GraphPad Prism8.3.0 (San Diego, CA) was used for statistical analyses. The Shapiro–Wilk test was used to assess data normality. Paired *t* -test was used to compare the means of HIF-1α with and without compressive force application. Student’s *t* -test was used to compare the means of HIF-1α expression among the genotypes in rs2057482 and rs2301113. Statistical significance was established at 5% (p<0.05).

## Results

### Evaluation of the expression of HIF-1α protein

The protein expression analysis showed that HIF-1α was significantly enhanced by simulated orthodontic compressive pressure application regardless of the genotype.

**Figure 2:**
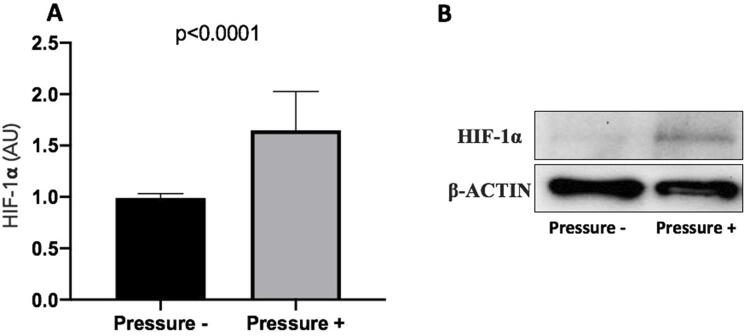
HIF-1α protein expression. A - Comparison of HIF-1α protein expression between no pressure (control, normalized to 1) and during compressive force application. Statistics: paired t-test. B - Representative immunoblot of HIF-1α protein expression. AU=arbitrary units

Figure 2 shows these results. The graph in Figure 2A shows the comparison of HIF-1α protein expression between no pressure (mean=0.99; Standard deviation=0.04) and during compressive force application (mean=1.64; Standard deviation=0.35). A statistical significance difference between the groups was observed (p<0.0001). Figure 2B is a representative demonstration of the immunoblot of HIF-1α protein expression.

### Evaluation of the influence of the genetic polymorphisms rs2301113 and rs2057482 on the expression patterns of HIF-1α protein

**Figure 3:**
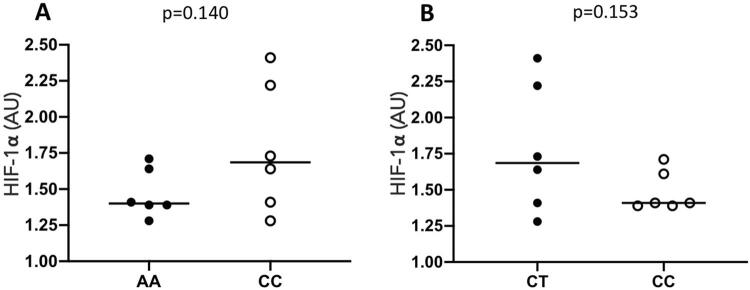
HIF-1α expression (bar indicates mean) according to the genotypes during compressive force application. A - Polymorphism rs2301113. B - Polymorphism rs2057482. AU=arbitrary units. Statistics: Student’s t-test

For the genetic polymorphism rs 2301113 (A>C), the mean and standard deviation of HIF-1α expression for the AA genotype was 1.470 (standard deviation 0.166), and 1.782 for the CC genotype (standard deviation 0.447). No statistical significance difference was observed between the homozygous genotypes AA and CC (p=0.140). Figure 3A shows the result of the comparison between genotypes for the genetic polymorphism rs2301113.

For the genetic polymorphism rs2057482 (C>T), the mean and standard deviation of HIF-1α expression for the CT genotype was 1.782 (standard deviation 0.447), and 1.487 for the CC genotype (standard deviation 0.138). No statistical significance was observed difference between the homozygous genotype CC and the heterozygous genotype CT (p=0.153). Figure 3B shows the result of the comparison between genotypes for the genetic polymorphism rs2057482.

## Discussion

Data from human research and animal experiments indicate that the rate of OTM changes according to the level and frequency and duration of applied force is influenced by the individual biological response of the periodontal ligament (PDL) and the alveolar bone.^21^ The variations of the response of the PDL and bone among individuals can be explained by some factors including the individual genetic background. The hypothesis that the genetic predisposition influences the individual response to OTM is supported by animal experiments and clinical studies in humans. In animals, individual variations of OTM rate are observed even with consistent orthodontic forces.^22 , 23^ This fact coincides with clinical observations in humans that led to the concept of “slow movers” and “fast movers”.^24^ For this reason, we evaluated the effect of two genetic polymorphisms in *HIF1A* on protein expression levels during simulated orthodontic compressive pressure to investigate if rs2301113 and rs2057482 are candidate genes for “slow” or “fast” movers.

The PDL and alveolar bone are two essential tissues for understanding OTM.^25^ hPDL fibroblasts play a major mediating role in the early phase of OTM with a differentiated and time-dependent regulation and expression pattern of many factors that will allow tooth movement.^6^ Thus, *in vitro* studies using hPDL cells are important tools to identify genes up- or down-regulated during OTM.^5 - 8^ These genes comprise many cytokines, which are involved in the aseptic inflammation that occurs in OTM, including the RANK–RANKL–OPG system, which is an essential signaling pathway during bone remodeling and the angiogenesis-inducing gene *VEGF-A* (Vascular Endothelial Growth Factor Alpha).^3 , 26^ Ullrich, et al.^8^ (2019) also identified that HIF-1α protein levels were significantly increased by simulated orthodontic compressive pressure irrespective of the oxygen supply. In our study, we also could confirm that HIF-1α protein levels increase during simulated orthodontic compressive pressure regardless of the genetic background of the sample.

VEGF-A is a growth factor involved in the remodeling of blood vessels and angiogenesis.^27^ The expression of angiogenic growth factors, such as VEGF-A, is induced by hypoxia via the transcriptional activity of HIF-1α.^28^ It is important to emphasize that during OTM the formation of new blood vessels and vasodilatation of existing blood vessels in the periodontal ligament occurs at pressure zones.^29^ Previous animal and *in vitro* studies suggested that the elevation of VEGF-A expression occurs in the very early phases of OTM.^6 , 29^ It is plausible to assume that the increased levels of HIF-1α observed here are also observed *in vivo* and followed by an increase in VEGF-A expression.

As previously stated *, in vitro* studies using hPDL cells are important to identify genes involved in the OTM, but also in the identification of candidate genes for future clinical studies that will aim to identify “slow movers” and “fast movers.”^5^ *In vitro* studies with human samples allow us to verify whether genetic polymorphisms may account for mRNA and protein expression and may affect the orthodontic clinical outcome. A previous study showed that a missense variant, which leads to a change of a proline to a serine, in the gene encoding *HIF1A* is associated with the expression of HIF-1α protein in breast cancer.^30^ However, our study did not support that variations in rs2301113 and rs2057482 play a role in protein expression in OTM and might be not involved in the genetic predisposition for “slow” or “fast” mover phenotypes. However, it is important to emphasize that we investigated genetic polymorphisms located in non-coding region, which do not encode protein sequences, different from the study performed by Kim, et al.^30^ (2008).

Our study has some limitations. It is possible that other genetic polymorphisms and mutations in *HIF1A* are involved in the variations in the level of the HIF-1α protein; however, the allele/genotype frequency of the other polymorphisms was an important limitation since our sample size per group was small and this is the main limitation of our study. Following a similar pattern, it is possible that the TT genotype in rs2057482 would differentially express HIF-1α compared to CC; however, in our sample, none of the cases present the double mutant homozygous TT. Thus, we used the heterozygous TT (carrying only one mutant allele) as a control. So, if this genetic polymorphism acts in a codominant fashion, it would be possible to observe a difference in the expression when comparing the heterozygotic samples with CT with the wild type homozygotic samples with CC. Nevertheless, if the genetic polymorphism rs2057482 acts in a recessive model, our study design would have been unable to detect the statistically significant difference for this variant since none of the samples were TT. An important limitation of the study design is the fact that, besides the analyzed SNPs, the patients’ genetic profile also varies, introducing confounding variables. Although this is a frequently used approach, only *in vitro* models with genetic constructs allow to prove the effect of a specific genetic variant in gene expression regulation.

Although a statistical association was not observed in our study, we cannot exclude a more complex genetic basis for the HIF-1α protein expression during OTM. In fact, this transcription factor was significantly increased during *in vitro* -simulated orthodontic compressive pressure and variation in its expression could be due to genetic polymorphisms and uncommon mutations in *HIF1A* or in other genes that regulate *HIF1A* . Further studies are necessary to identify candidate genes for “slow movers” and “fast movers” during orthodontic treatment.

## Conclusion

Our study confirmed that compressive pressure application enhances expression HIF-1α protein expression. However, we could not prove that the polymorphisms rs2301113 and rs2057482 located within protein-noncoding *HIF1A* gene affect the expression patterns of HIF-1α protein by hPDL fibroblasts during simulated orthodontic compression. These results suggest that rs2301113 and rs2057482 are not candidate genes for clinical studies in orthodontic research; however, other polymorphisms in *HIF1A* gene could be involved in HIF-1α protein expression by hPDL fibroblasts during simulated orthodontic force.

## List of abbreviations

OTM: Orthodontic Tooth Movement
HIF1A: Hypoxia-inducible factor α-subunits
HIFα: Hypoxia-inducible factor α-subunits
hPDL: Human periodontal ligament
PDL: Periodontal ligament
RANKL: Receptor activator of nuclear factor-κB ligand
COX2: Cyclooxygenase-2
VEGF-A: Vascular Endothelial Growth Factor Alpha
PCR: Polymerase chain reaction
PVDF: polyvinylidene difluoride
TBS-T: Tris-buffered saline and 0.1% Tween 20, pH 7.5
